# American Institutions for the Insane

**Published:** 1852-10-01

**Authors:** 


### AMERICAN INSTITUTIONS FOR THE INSANE.

We subjoin an able article from the pen of a distinguished American psychologist, Dr. Pliny Earle, on the present state of the principal Asylums for the Insane in the United States of America.* It is taken from two consecutive numbers of Dr. Hay's ably conducted and excellent periodical, *The American Journal of the Medical Sciences*. The reports upon which this paper is based are so difficult to obtain in this country, that we are glad of an opportunity of placing this abstract before our readers, all of whom are much interested in the progress of medico-psychology among the enlightened members of our profession engaged in the treatment of the insane on the other side of the Atlantic.

More than two years have elapsed since we last published an abstract of the reports emanating from the various public institutions for the insane, then existing in the United States. During the intervening period, the general scheme for meliorating the condition of those who are afflicted with mental disorders, has advanced with that accelerated rapidity, imparted to it by the active and energetic labours of the preceding twenty years. Two new State institutions have gone into operation; several of those previously existing have been enlarged, and measures have been taken by the legislatures of other States, for the erection of similar establishments within the limits of their legislation, respectively. Miss Dix, whose labours in the cause are too generally known to require a recapitulation, has pursued her mission with an energy that never abates, and an assiduity that knows no interruption. The medical superin-

* "Reports of American Institutions for the Insane":—1. Of the Maine Insane Hospital, for 1848, 1849, and 1850. 2. Of the New Hampshire Asylum, for 1849 and 1850. 3. Of the McLean Asylum, for 1849 and 1850. 4. Of the Massachussets State Hospital, for 1849 and 1850. 5. Of the Butler Hospital, for 1849 and 1850. 6. Of the Hartford Retreat, for 1848, 1849, and 1850. 7. Of the Bloomingdale Asylum, for 1849 and 1850. 8. Of the New York State Lunatic Asylum, for 1849 and 1850. 9. Of the New York City Asylum (Blackwell's Island), for 1849 and 1850. 10. Of the New Jersey State Asylum, for 1849 and 1850. 11. Of the Pennsylvania Hospital for the Insane, for 1849 and 1850. 12. Of the Frankford Asylum, for 1848, 1849, and 1850. 13. Of the Maryland Hospital, for 1846, 1847, 1848, 1849, and 1850.

tendents of several of the institutions have been changed. No less than three of the physicians who had become the most distinguished in this speciality of the profession have deceased. The aggregate number of the insane collected into hospitals has very considerably increased; and, finally, a large amount of matter has accumulated upon our hands. We proceed to lay such portions of this as we think the most interesting or useful before our readers:—

| | Men. | Women. | Total. |
|---|---|---|---|
| 1. The number of patients in the Maine Insane Hospital, March 31st, 1848, was . . . | 80 | 47 | 127 |
| Admitted during the year . . . . . | 60 | 63 | 123 |
| Number of cases under treatment . . . | 140 | 110 | 250 |
| Discharged during the year . . . . | 61 | 62 | 123 |
| Remaining March 31st, 1849 . . . . | 79 | 48 | 127 |
| Of those discharged, there were recovered . . | 11 | 17 | 28 |
| Died . . . . . . . . . | 8 | 10 | 18 |

*Causes of Death.*—Dysentery, 9; phthisis, 4; apoplexy, 2; "exhaustion," 2; partial paralysis, 1.

"About the middle of August," says the report, "with more inmates than at any (previous) time since the house was erected, a malignant dysentery, or rather *colonitis*, began to afflict our inmates, and, soon after, several of the officers and attendants were prostrated by it. In a few weeks, about fifty cases occurred, of whom nine died. Severe and unmanageable as the disease showed itself, one death only took place in any person who had not been weakened by years of previous disease. If the disease was not *rheumatic* in its character, it certainly was followed, if not suspended, by an acute type of that malady, affecting the membranes of joints, and in one case, the joints and eyes alternately. There were but few cases of relapse. In one, however, there was a renewed attack and death, after several weeks of convalescence. * * *

"We are constrained to confess that the common remedial agents disappointed our expectations; and were the disease to repeat its visit, with the knowledge thus far acquired, we should confine our action to cleanliness, ventilation, and the administration of such quieting or stimulating remedies as nature seemed to require, to enable it to struggle through the contest. Few diseases, if any, are accompanied by more offensive emanations than the one under consideration."

Before reading the report, we do not recollect to have met with the result of any researches in regard to the comparative curability of suicidal insanity and other forms of the disease. Hence, we regard the following extract as one of no inconsiderable interest. "Of eight hundred and sixty-eight cases which have been in this hospital, *one hundred and one* are known to have had a propensity to suicide. *Fifty-one* have recovered, which is *twelve per cent.* greater than the average recoveries on eight hundred and sixty-eight admissions. Whether this results from the disease being more easily recovered from, or in consequence of their being sooner committed, I have not the means of judging with any degree of accuracy. It is certain that some of the most perfect recoveries that ever took place here, happened to some of the most determined of this class."

On the 1st of January, 1849, an additional edifice, forming a wing one hundred and fifty-eight feet in length, and designed for male patients, was opened for occupation. It was warmed by steam, according to the present improved method. Dr. Bates, in this report, recommends the construction of a similar wing for females, to meet the increasing applications for the admission of patients.

The report for 1849, opens with an allusion to the exemption of the inmates of the establishment from the Asiatic cholera, which prevailed in the summer of that year.

| | Men. | Women. | Total. |
|---|---|---|---|
| Patients, March 31st, 1849 . . . . . | 79 | 48 | 127 |
| „ admitted during the year . . . | 63 | 63 | 126 |
| Whole number of cases „ „ . . . | 142 | 111 | 253 |
| Number discharged „ „ . . . | 56 | 53 | 109 |
| „ remaining March 31st, 1850 . . | 86 | 58 | 144 |
| Of patients discharged, there were recovered . | 30 | 28 | 58 |
| Died . . . . . . . . . . | 8 | 7 | 15 |

*Causes of Death.*—Paralysis, 4; marasmus, 4; apoplexy, 2; exhaustion, 2; phthisis, 1; phrenitis, 1; old age, 1.

The *piquancy* of the following extract is peculiarly refreshing. "Whether more or less of our patients recover than in other institutions, exactly similar in all respects, if there be such, I am unable to say. Some boast of more cures than have ever been realized here, while others, equally meritorious, so far as I know, have been so modest as to report even less than we have done. We are certain that many of our institutions possess appliances which are not, and probably never will be furnished to this; and it is not unreasonable, other things being equal, to look to them for greater success."

As a suitable companion to the foregoing, we subjoin the doctor's remarks upon statistics. "When honestly made, they are not likely to do injury; but I am sure they are sometimes made instruments of deception. If figures cannot lie, they may mislead, by disguising the truth. For instance; suppose, at the end of each year, instead of reporting all cases as *recent*, which were actually admitted within one year of the attack, I should, for the purpose of *appearing* to cure 90 per cent. of recent cases *discharged*, report only *such* as *recent* cases as had not become *old* ones by remaining with us, I might impose the belief on the *uninitiated*, that 90 per cent. of recent cases could be cured; when every man acquainted with the subject knows, that no instance can be shown, in which 90 out of 100 cases, admitted in succession, no matter how *recent*, were ever cured.

"On our examination of our records, I find there remain, this day, sixty-five cases which were admitted within one year of the attack . . 65

| | |
|---|---|
| Seventy-nine which were more than one year . . . . | 79 |
| Total . . . . . . . . . . . . . | 144 |

"But 'as the manner of some is,' calling none *recent*, except such as have not *now* been insane over one year, the account stands,

| | |
|---|---|
| *Recent* (cases) . . . . . . . . . . | 36 |
| Old „ . . . . . . . . . . . | 108 |
| Total . . . . . . . . . . . . . | 144 |

Our cases remain as first recorded."

Few physicians have entered more into the details of the statistics of insanity than Dr. Bates, and his productions in this department carry with them the evidence of "honesty" in their compilation, while their accuracy and perspicuity are unrivalled.

The value of many of the statistics of insanity is materially deteriorated by including the whole number of *cases* rather than that of the number of *persons* admitted into the hospitals. The second and still subsequent receptions of the same individuals, form an element in the calculation too important to be overlooked. Thus, according to the report before us, the whole number of *cases* admitted into the hospital was 994, while the number of *persons* was but 807. The admissions after the first were as follows:—Second 134, third 26, fourth 14, fifth 6, sixth 3, seventh 2, eighth 2.

Of the 994 *cases*, 403 had been discharged cured, and 78 had died.

In the course of the last winter, the public were generally informed, through the medium of newspapers, of a terrible incident in the history of the Maine Insane Hospital. In their report for 1850, the trustees of that institution thus allude to the fatal accident.

"Since the last annual report, the two south wings of the hospital have been burnt. . . . . . This catastrophe occurred between three and four o'clock on the morning of December last (1850)—a period when all was quiet, and supposed to be secure from any casualty of this sort. The fire originated in the hot air-chamber under the old south wing, probably from some defect in the arrangement of the smoke-pipe connected with the warming apparatus, and spread with great rapidity. The flues leading from the hot air-chamber, affording a direct communication, very quickly filled the galleries with smoke, gas, and heat, incompatible with human life, rendering it more than probable that those who perished were suffocated long before the fire reached them.

"Soon as the fire was discovered, every effort was made for the rescue of the inmates; first, by opening their dormitories, and, when the smoke and gas rendered that impracticable, recourse was had to the windows on the outside of the building, by means of ladders, by which several were removed.

"The progress of the fire was checked before it reached the north wing, consequently the female patients were all safely removed.

"Twenty-seven of the inmates (patients) perished in the conflagration. One of the attendants, Mr. H. D. Jones, while nobly exerting himself to rescue the patients, shared the same fate.

"The officers, without much difficulty, succeeded in procuring good temporary accommodations for the inmates, in private dwellings, in the Augusta House, and, for some of the most furious, in the county jail, under the immediate supervision of the attendants, until they could be removed by their friends, or otherwise provided for."

Dr. Bates, in his accompanying report, says, "To those who merely speculate on such matters, it may be easy to conjecture how fire should be communicated from a funnel sixty feet from the fire-grate, when no fire had been placed in the furnace for more than nine hours; but to those (a jury of inquest) who spent ten days in the inquiry, it remained a matter of uncertainty. The stove-pipe, near where the fire was first discovered, had been put up under the steward's directions, within six weeks of the time of the fire; and, though I never examined that portion of the pipe, I had every reason to believe that it occupied the same position it did during the whole of the winter previous.

"Much has been said about the fire being set by an incendiary; some undoubtedly believe it now. It may not be improper for me to say, I have never entertained such an idea for a moment."

| | Men. | Women. | Total. |
|---|---|---|---|
| Number of patients March 31st, 1850 . . | 86 | 58 | 144 |
| „ admitted in 10 months, to Jan. 31, 1851 | 50 | 40 | 90 |
| Whole number . . . . . . . . | 136 | 98 | 234 |
| Discharged . . . . . . . . . . | 104 | 78 | 182 |
| Remaining January 31st, 1851 . . . . | 32 | 20 | 52 |
| Of those discharged, there were cured . . | 30 | 21 | 51 |
| Died . . . . . . . . . . . | 35 | 4 | 39 |

*Causes of Death.*—"Suffocated at the burning of the hospital," 27; general paralysis, 3; marasmus, 3; "prostration, from violent mania, 2; phthisis, 1; old age, 1; chronic abdominal inflammation," 1; acute inflammation, 1.

The people of Maine are indebted to Dr. Bates for the original draft of their laws in regard to the insane, and to insanity in all its legal relations. These are, undoubtedly, as nearly perfect as any in existence. In the report before us, he makes the proposition subjoined.

"I submit, for the consideration of the legislature, the subject of a penal act, making it the duty of any person who shall know of any cruelty or abuse to an insane person in the Insane Hospital, or elsewhere in this State, to give information thereof to a magistrate, or to the superintendent (if at the Hospital), within ——— days."

In January last, Dr. Bates, having been appointed by the Governor and Council to visit the Institutions in other States, for the purpose of learning any improvements which may be introduced when the Maine Hospital shall be re-built, resigned the place of superintendent. Dr. H. M. Harlow, the Assistant Physician, has hitherto fulfilled the duties of the position. He makes an addition to this report for the two months necessary to complete the official year.

| | Men. | Women. | Total. |
|---|---|---|---|
| During this period there were admitted . . | 6 | 7 | 13 |
| ,, ,, ,, discharged . . | 6 | 3 | 9 |
| ,, ,, ,, cured . . . | 3 | 1 | 4 |
| ,, ,, ;, died . . . | 2 | .. | 2 |
| Remaining March 31st, 1851 . . . . . | 34 | 24 | 58 |

*Causes of Death.*—Serious apoplexy, 1; phthisis, 1,

In some remarks upon hereditary insanity, Dr. H. says, "it is, *undoubtedly*, a fact that the mother is more likely to transmit the predisposition than the father, and a good deal more likely to transmit it to daughters than to sons; while the father more frequently transmits it to the sons." These propositions are asserted with a degree of positiveness which, in the present state of knowledge upon the subject, appears to us as hardly warrantable.

In regard to the prevalence of mental disorders, he says, "we hazard the opinion that, could an accurate census be taken of the insane and idiots who are incapable of taking care of themselves, in Maine, the proportion would be found to be one in every three hundred of its inhabitants. And the same melancholy fact, we believe, would obtain in all the other New England States." We do not recollect to have seen so high an estimate of the insane and idiots in any other portion of the world, excepting, perhaps, a part or the whole of Scotland, and a district in Yorkshire, including the city of York, England.

2. "So far as statistics can furnish an inference," says Dr. McFarland, of the New Hampshire Asylum, the past year (1849) has been the most successful that the institution has known since its foundation. A number unusually large has been received, more have recovered, and the proportionate mortality has been less than in any preceding year.

| | Men. | Women. | Total. |
|---|---|---|---|
| Number of patients, May 31st, 1849 . . . . | 52 | 62 | 114 |
| ,, admitted during the year . . . . | 59 | 44 | 103 |
| Whole number . . . . . . . . . | 111 | 106 | 217 |
| Discharged . . . . . . . . . | 38 | 52 | 90 |
| Died . . . . . . . . . . | 3 | 4 | 7 |
| Remaining, June 1st, 1850 . . . . . | 69 | 58 | 127 |
| Of those discharged, there were cured . . . . | 17 | 28 | 45 |

The report states, that the institution has "been filled with inmates beyond its proper capacity during the whole of the official year. To remedy this inconvenience, and to meet the increasing necessities of the public, the Legislature of the State made an appropriation, in July, 1849, for the purpose of erecting an additional wing for the accommodation of fifty patients. This edifice, at the time of the report nearly finished, is one hundred and twenty-six feet long, thirty-six wide, and three stories high above the basement. It is intended for men.

We select the following passage, not only for the truths which it contains, but also for the beautiful style in which it is written.

"It has always been a striking feature in the whole subject, that every truly well constructed and well managed asylum for the insane, in this country, has always been filled with inmates. There is no such thing as *properly* managing the insane, *as a class*, elsewhere, without a cost beyond the means of most in a community like ours. So far as kindness of treatment and safety of person are concerned, there is no doubt that the odds are decidedly in favour of collecting the insane into hospitals. Money may purchase proper attention at home; kindred and affection will never, save in extraordinary instances, render it: they are ties which, sooner or later, give way. *Incurable insanity is but the half-finished work of death.* The destroyer has swept away all save the unhumanized shape, around which affection will not for ever linger. This is a truth towards which all experience leads; and its universal recognition, among the philanthropic in both hemispheres, has created the lunatic asylum, now an indispensable part of the machinery of human society.

"It is no violation of the principles of duty and affection, that the living should seek to bury the dead from sight; neither should any false conception of the obligations owed the insane require, that those whose services society demands should remain for ever in the exhausting contemplation and the vainly attempted preservation of ruins hardly less abhorrent than those consigned to the dust, after the extinction of all vitality.

"The first and best efforts should be used to effect restoration. Failing in that, it is no small boon to avert the progress of mental decay, and throw around the unfortunate a shield from an exposure at which every sentiment of propriety and humanity revolts."

We have rarely, if ever, seen so faithful a picture of the mental and moral position of the medical officer of an establishment for the insane, as that with which this report closes. As it occupies but little space, we present it without curtailment.

"It is no trivial matter to assume the right to think and act for a body of our fellow-beings, of whose liberty we have become custodians, and whose minutest movements we may be obliged to direct. It is no irresponsible undertaking to impose restraints which, if protracted, may be injurious, or to grant liberties which, if transcended, may prove fatal. It requires no little schooling of the sensibilities to listen patiently, for the hundredth time, to the complaint which has no existence save in the disordered fancy; to parry the request that cannot be granted, and which it is painful to deny; to frame a new reply to the interrogatory that has been and will be repeated with every meeting, however often; to meet the eye, whose every glance is a volume of yet unexpressed suffering, that admits no mitigation. It needs more than human aid to keep unexhausted the fountains of sympathy, and to bear cheerfully a burthen from which night affords no relief. If to walk daily amid scenery like this is the ordinary lot of all who assume the care of the insane, under the most happy circumstances, the case puts on a new aspect, if to this be added the never yet described condition of a *crowded* lunatic asylum. There is then a painful sense of irritation read in every movement and feature of those who are seeking vainly, amid the throng, the disbursement of an overcharged brain, in solitude and silence. Suffering, as it is reflected from one countenance to another, re-creates itself, and each reduplication is clothed with new horrors."

| | Men. | Women. | Total. |
|---|---|---|---|
| According to the last report of Dr. McF., the number of patients in the Asylum, May 31, 1850, was | 69 | 58 | 127 |
| Admitted during the year . . . . . . | 44 | 44 | 88 |
| Whole number admitted during the year . . | 113 | 102 | 215 |
| Discharged during the year . . . . | 54 | 44 | 98 |
| Remaining May 31, 1851 . . . . . . | 59 | 58 | 117 |
| Of those discharged, there were cured . . | 31 | 14 | 45 |
| Died . . . . . . . . . . | 2 | 10 | 12 |

*Causes of Death.*—"Insanity of advanced age," 5; epilepsy, 2; phthisis, 2; dysentery, 1; typhoid fever, 1; marasmus, 1.

The report before us is written with much ability, and is unusually interesting. As our limits deny us the pleasure of extracting all that we might wish, we must confine ourselves to such portions as appear to us of paramount importance.

"The system of lodging patients in associated dormitories, which is adopted in part (in the new building mentioned in the report for 1849), operates favourably beyond our anticipations. It is not contended that the insane, as a body, would be safe, associated with no discrimination, save of sex, in a common sleeping-room. Yet many, either from timidity on their part, or as a protection against self-injury, are more properly lodged in that manner."

The following remarks are valuable in their bearing upon medical jurisprudence.

"It is the most nice point in all the departments of philosophy, to ascertain precisely what effects a given amount of mental impairment will exhibit; or, in other words, to predicate, upon the language, actions, and other general demeanour of an individual, how unsound his intellect may be. This uncertainty obviously lies at the foundation of all the difficulty in fully establishing the legal relations of the insane. The experience gathered here shows that when we thus reason from effects to causes, *we most frequently set the amount of actual disease too low, and that events sooner or later teach us that the degree of mental unsoundness is greater than we anticipated. Instances by the score could be gathered from our case-book, to substantiate this position. Many have been fit inmates, whose appearance and address would have staggered a court of justice, if called on to decide the existence of insanity.*"

It appears that, in New Hampshire, there are no statutory enactments requiring a legal examination of the mental condition of an individual, previous to his committal to the asylum. The friends of the patient may alone assume the responsibility of thus depriving him of his liberty. The dangers of this state of things are well portrayed in the report, and illustrated by appropriate cases. The basis of a code of laws is then proposed, which would cover the whole ground of the subject, providing for a careful pre-examination of every case of alleged insanity, defining the powers of magistrates and of courts of probate and judicature, in regard to the insane, protecting the asylum, its officers, and the friends of the patients in case of committal, carefully guarding the rights of the patient, defining his responsibility in business transactions, and in civil and criminal suits, his testamentary ability, &c. &c.

In the summer of 1850, Dr. McFarland visited about twenty of the institutions for the insane in England, Scotland, France, and Italy. Some of the results of his observations are embodied in this report, from which we make the subjoined extracts.

"A visitor to the English and French hospitals is immediately struck with the great evident cost of many of them, compared with the number they are intended to receive. This is no test, however, of their excellence, which, architecturally considered, lies in their spaciousness, the altitude of their ceilings, and the strict attention paid to the details of heating and ventilation. The gloomy interior of most of the American asylums, where the light must be excluded by a mischievous and false economy, finds no parallel in Europe, save in the extremely old institutions of the North, or those of Catholic countries, where an asylum is most frequently a suppressed monastery.

"We (Americans) do not suffer in the comparison (of institutions). Indeed, there is much reason for self-gratulation. Our institutions are better organized; and, if our edifices for the reception of lunatics be not so spacious, we are already alive to their deficiencies, so that there is no obstinate adherence to exploded designs. We have no mischievous precedents, gray with age, to be annihilated. We have no evils to anticipate, like those which hang their weight upon the charities of communities, who, in a thousand other ways, are now paying the debts imposed by the usages of barbarous times.

"Insanity in America is ever presenting to us almost precisely the same aspects. In very old communities, where the lines between different grades of society have been closely drawn for ages; and where contiguous neighbourhoods, from different pursuits, have a distinct character, in no place is the difference more quickly seen than in the lunatic asylum. While the lunatic of Louisiana is almost of the same mould with him of Maine, the plodding agricultural serf of the North Riding of Yorkshire seems, when insane, a totally different being from the coal miner of Durham, or the manufacturer of the West Riding, and each neighbours of but an hour's journey removed."

3. Dr. Bell, in the report of the McLean Asylum, for 1849, says: "The cholera, in its visitation to this section of New England, held one of its strong holds just across our border line; and many of the most intense and virulent cases of this fell epidemic were in the village between us and the city. Yet we were wholly preserved; if there were premonitory indications of the effects of a malarious atmosphere among us, their actual nature was lost sight of in the facility of their yielding to medical agents."

| | Men. | Women. | Total. |
|---|---|---|---|
| Number of patients at beginning of year | 77 | 84 | 161 |
| Number admitted during the year | 77 | 83 | 160 |
| Whole number " " | 154 | 167 | 321 |
| Discharged " " | 59 | 78 | 137 |
| Remaining at end of year | 95 | 89 | 184 |
| Of those discharged, there were recovered | 26 | 38 | 64 |
| Died | 9 | 6 | 15 |

The institution was filled to its utmost capacity for patients during the whole year.

"We have resorted," says the report, "to personal restraints only in some two or three cases, where it was believed that life could not have been preserved without a resort to such aid."

During the thirteen years that Dr. Bell has been connected with this asylum, 1857 patients have been admitted, of whom 948 have recovered, and 199 have died.

From the report for 1850, we gleam the following items:—

| | Men. | Women. | Total. |
|---|---|---|---|
| Patients at beginning of the year | 95 | 89 | 184 |
| Patients admitted during the year | 80 | 93 | 173 |
| Whole number admitted during the year | 175 | 182 | 357 |
| Discharged | 75 | 82 | 157 |
| Remaining at end of year | 100 | 100 | 200 |
| Of those discharged, there were cured | 34 | 44 | 78 |
| Died | 15 | 13 | 28 |
| Patients admitted from 1837 to 1850, inclusive | .. | .. | 2030 |
| Of whom have been cured | .. | .. | 1026 |
| Of whom have died | .. | .. | 227 |

During this period, the average number of patients, annually resident, has gradually augmented from 80 to 201.

By the following extract, it will be perceived that this institution has attained an exemption from one of the most discouraging obstacles with which the physicians of such establishments are generally obliged to contend.

"What was, a dozen years since, one of the most painful and disheartening circumstances in the experience of those in charge—the capricious removal of patients at the most critical and promising stages of restoration, soon to fall back into permanent disease, is now a rare occurrence." This desirable condition of things has been effected principally by a fund contributed by the Hon. William Appleton, the proceeds of which are devoted to defraying the expenses of those patients whose pecuniary means, and those of their friends, are such as to require this assistance. A worthy example, this, to be followed by the wealthy! Nor is this the only instance of the benevolence of the same generous donor. "The recent decision," says the report, "of our munificent friend, the Hon. William Appleton, to continue his course of liberal benefactions to our institution, by bestowing upon us the means (*twenty thousand dollars*) of establishing two distinct edifices, in the neighbourhood of the other buildings, for the accommodation of a class of patients most favoured by fortune, with arrangements more extensive, complete, and commodious than have been before known in this or perhaps any other country, will be an era in the history of the asylum."

A billiard-room, fifty feet long by twenty-five wide and fourteen high, was constructed during the past year; and heating by water, with forced ventilation, has recently been introduced into the whole establishment.

We close the notice of this report with the remarks of Dr. Bell upon the importance of well-endowed institutions.

"As the communities called to provide for the insane advance in familiarity with this duty, and in means to meet it, the fatal error of *cheap* institutions will cease to exist; an error involving not merely the negative objection of leaving the presumptive ends of hospital treatment unfulfilled, but the positive hazard of accidents, compromising not only the institution immediately concerned, but the usefulness and reputation of the whole class. It would be a happy conviction upon the minds of legislators and communities, could they be persuaded that, between no provision at all of a public kind for the insane, and a parsimonious, stinted, and inefficient imitation of a real provision, the former evil is infinitely the least. A county, or town, or state, may dignify a part or the whole of some custodial receptacle for its lunatics, with the high-sounding title of an 'Asylum;' the public and curators of the unfortunate, or even the friends and relatives, may ignorantly, or as an excusing salvo, accept such substitution as a full acquittance of their obligation; but every person who gives an hour's reflection to the matter, and compares the cost of persons in health, and of the insane under even the minimum outlay for mere custody, to say nothing of amelioration and cure, cannot but see the impossibility of doing justice to the insane on a cheap plan."

4. The report of the Massachusetts State Lunatic Hospital, for 1849, says: "The hospital has been more crowded the past year than ever before. The extent of its accommodation does not exceed what three hundred and seventy-five requires. At no time has there been less than four hundred and five patients. The greatest number was four hundred and forty. The average for the year about four hundred and twenty."

| | Men. | Women. | Total. |
|---|---|---|---|
| Patients at beginning of the year | 217 | 192 | 409 |
| Patients admitted in course of the year | 134 | 139 | 273 |
| Whole number admitted in course of the year | 351 | 331 | 682 |
| Discharged in course of the year | 131 | 122 | 253 |
| Remaining at the end of the year | 220 | 209 | 429 |
| Of those discharged, there were cured | 70 | 68 | 138 |
| Died | 19 | 18 | 37 |

"The diseases usually prevalent in the warm season," writes Dr. Chandler, "prevailed to some extent among our patients and their attendants. Diarrhœa, dysentery, fever, and a few cases of the graver forms of cholera morbus, and cholera, with all its characteristic features, occurred among our household in the month of August. By strict and immediate attention to the first indications of diarrhœa and the forming stage, only eleven cases, all of which were among the male patients and their attendants, took on the more severe and unmanageable symptoms of cholera. Four died very suddenly of this mysterious scourge. Three of them had become debilitated by long and incurable disease, and the fourth, although he was fleshy and laboured much in the open air, was in the habit of drinking enormous quantities of cold water. All through the summer, we took the precaution to place fires in all the furnaces whenever the weather was cool or damp.

"It is somewhat remarkable that the inmates of this hospital should be almost entirely free from all bowel complaints until about the first of August; that these diseases should then commence and become more and more prevalent and more fatal up to the third of September, and that they then should suddenly cease as an epidemic. Since that time we have been happily relieved of any great amount of sickness among our patients; but there have been several cases of typhoid fever among the attendants.

"On the 19th of March, one of our attendants became sick with the measles. Three successive *crops* of this disease succeeded. Thirteen attendants, eight patients, and my two daughters had it. The last of the fourth crop became sick on the 30th of April following. It was noticed that the attendants—those who were supposed to be in better health than the patients, and who were capable of taking more rational care of themselves, had the disease, almost uniformly, in a more severe form, and apparently suffered more from it than the patients. In the forming stage of the disease, the patients lived in a more uniform temperature, and were less exposed to the vicissitudes of the season than the attendants."

Cases of mental improvement, caused by attacks of insanity, have heretofore been recorded by several writers. Dr. Chandler says: "I have known a few individuals who were brought here insane, and who recovered to become better citizens than they were before. Their minds and feelings acquired strength and soundness by the disease and by undergoing the process of cure, as some musical instruments are said to be improved by being broken and repaired again.

| | Men. | Women. | Total. |
|---|---|---|---|
| By the report for 1850, it appears that the number of patients remaining December 1st, 1849, was | 220 | 209 | 429 |
| Admitted during the year . . . . . . | 129 | 112 | 241 |
| Whole number . . . . . . . | 349 | 321 | 670 |
| Discharged . . . . . . . . . | 120 | 109 | 229 |
| Remaining November 30th, 1850 . . . | 228 | 213 | 441 |
| Of those discharged, there were cured . . . | 60 | 65 | 125 |
| Died . . . . . . . . . . | 29 | 28 | 57 |
| The whole number admitted from 1833 to 1850, inclusive, a period of 18 years, is . . . | 1818 | 1780 | 3598 |
| Of whom have been discharged, recovered . . | 818 | 876 | 1694 |
| Died . . . . . . . . . . | 199 | 167 | 366 |

*Causes of Death.*—Marasmus, 61; apoplexy and palsy, 43; consumption, 39; epilepsy, 38; disease of heart, 18; suicide, 17; disease of brain, 17; typhus fever, 10; lung fever, 12; hemorrhage, 5; dysenteric fever, 8; cholera morbus, 4; inflammation of the bowels, 4; mortification of limbs, 3; dropsy, 6; chronic dysentery, 4; erysipelas, 12, diarrhœa, 16; diseases of brain from intemperance, 2; bronchitis, 3; old age, 5; gastric fever, 4; land scurvy, 1; congestive fever, 2; concussion of brain, 1; disease of bladder, 1; fright, 1; rupture, 1; exhaustion, 19; convulsions, 2; cholera, 4; asthma, 1; hydrothorax, 1; cancer, 1.

The report says, that, during the last year, "we have had nothing like an epidemic, unless about twenty-five cases of erysipelas, which occurred in the spring, may be so called. These cases made their appearance from the last of February to the first of June in a majority of the wards, without being in any instance contagious. No cause can be assigned with any certainty for their breaking out then more than at any other time. The inflammation was, in a majority of cases, confined to the head and face, and when the disease extended to the body it was apt to be fatal." Five patients and one female assistant died of it. "It was noticed that those patients who occupied rooms nearest our hot-air furnace, and were consequently the warmest, were most liable to its attack."

Epilepsy "is very often one of the prominent symptoms (attendants?) of insanity brought on by habitual intemperance; and where it is so, fatal results follow in a short time."

The table subjoined exhibits some not unimportant facts in relation to twenty-eight epileptic patients who have died in this hospital.

| | Average insanity before admission. | Average residence in hospital. | Average age at death. |
|---|---|---|---|
| 23 men . . . . | 36 months. | 13½ months. | 42 years. |
| 5 women . . . . | 60 ,, | 14 ,, | 38 ,, |

"The number of males afflicted with epilepsy in this hospital is greater than that of females. The males died at the most advanced age, but they may have been, and probably were, attacked with epilepsy later in life than the females.

"The accession of the fits of epilepsy are very irregular as to time and severity in different persons. Some have one or two fits every day or two. Some have ten or twenty in quick succession, and are much disturbed in mind for several days, to be followed by an interval of some weeks or months of freedom from fits, and by serenity of mind. Some are seized only while asleep, and others only while awake. In some, the fits amount only to slight dizziness which hardly takes away consciousness. In others, all the senses are locked up for the time, and the physical system is racked with convulsions horrid to behold. As a general thing, these persons are unconscious at the time of the fit, and, after apparently suffering the most frightful tortures, wake up and inquire of those around them what has happened. A very few have a short warning of the coming on of a fit, but generally they know nothing of it except as they are told by others. Most epileptics enjoy the pleasing delusion, that their fits are constantly becoming lighter and more unfrequent.

"The management of them should be kind and conciliating. About the time of having fits they are irritable, jealous, and easily provoked to violent actions. They will not be driven, but must be flattered. They should have exercise, but should never get fatigued. Their diet should be sparing, but nutritious. They should never overload the stomach or become surfeited.

"But little can be done in the way of medical treatment. In slight cases, stramonium, nitrate of silver, and sugar of lead have some reputation. In a few cases, *unconnected with insanity*, a mitigation and a cure even have followed their protracted use."

Heating by steam has been introduced into a part of the establishment, and lighting by gas into the female department.

The average number of patients, during the last year, was 440.

5. From the second annual report of the Butler Hospital for the Insane, at Providence, R. I., we learn that the number of patients at that institution:—

| | Men. | Women. | Total. |
|---|---|---|---|
| January 1st, 1849, was . . . . . . | 56 | 44 | 100 |
| Admitted in the course of the year . . . | 42 | 51 | 93 |
| Whole number admitted in the course of the year . | 98 | 95 | 193 |
| Discharged in the course of the year . . . | 47 | 39 | 86 |
| Remaining, December 31st, 1849 . . . | 50 | 57 | 107 |
| Of those discharged, there were cured . . | 24 | 11 | 35 |
| Died . . . . . . . . . . . | 11 | 9 | 20 |

*Causes of Death.*—Dysentery, 4; acute mania, 4; chronic mania, 7; epilepsy, 1; disease of heart, 1; abscess, 1; intestinal perforation, 1; pulmonary disease, 1.

"I need hardly say," remarks Dr. Ray, "that a general summary of results like this conveys but a very inadequate idea, to most persons, of the amount of good accomplished in a single year, by a hospital for the insane. To how few can the simple statement that so many have recovered, give any idea of the peculiar joy experienced by those who have seen the cloud of disease lifted from their spirits, and the undimmed light of reason shining serenely out upon their mental horizon! The hours of mental torture that have been soothed, the crushing burden of distrust and apprehension that has been lightened, the joy of those—the husband, father, child—who welcome the return of the loved one as from the grave; the relief of that desperate agony, which day after day has been aggravated by the appalling sights and sounds that often crowd upon the shattered mind, the restoration to the domestic circle of peace, order, and quiet, that has followed the withdrawal of some uneasy spirit, whom none of the arts of kindness could please or soften—these are benefits that cannot be estimated by figures, though not among the least conferred upon a community by establishments like ours. Neither are words more adequate to the purpose, because those benefits lie too far beyond the range of ordinary experience to be conceived of by any who have not personally seen and felt them.

"It will be noticed that four of the deaths were produced by dysentery. In this, as in many other parts of the country, cholera was immediately succeeded by dysentery, which prevailed with a degree of severity not experienced for many years. The former we fortunately escaped altogether. From the latter, however, no advantages of diet, ventilation, or cleanliness could entirely save us, although they probably rendered the disease of a milder character than it presented in the neighbourhood. The whole number of cases was nearly forty, besides several among our attendants and domestics."

Dr. Ray remarks that he believes the proportion of foreigners among his patients is much larger than it is in the same population, that they are less curable than Americans, and that this fact has been observed in other institutions. He alludes to the difficulties in treating them, and appears to approve of the construction of hospitals specially intended for them. He thinks the difficulties in treating them arise, at least in some measure, from the "inability to approach them in a proper way." We infer from his remarks, that he would have the officers of the special hospitals referred to foreigners also, or at least, more intimately acquainted with the language, idioms, modes of expression, manners, customs, and religious faith of the patients. We have no doubt that great advantages would result from such an arrangement, but the obstacles in the way to its attainment are various and great.

| | Men. | Women. | Total. |
|---|---|---|---|
| According to the report for 1850, the number of patients at the beginning of the year was | 51 | 56 | 107 |
| Admitted during the year | 38 | 35 | 73 |
| Whole number | 89 | 91 | 180 |
| Discharged | 38 | 29 | 67 |
| Remaining, December 31st, 1850 | 50 | 63 | 113 |
| Of those discharged, there were cured | 12 | 7 | 19 |
| Died | 7 | 9 | 16 |

*Causes of Death.*—Acute mania, 4; chronic mania, 4; meningitis, 3; "Bell's disease," (typhoid mania?) 1; consumption, 1; epilepsy, 1; disease of heart, 1; general paralysis, 1.

"Many of those who died had gradually approached the extreme limit of life, and ceased to exist less in consequence of any particular organic lesion, than *that gradual consumption of the vital forces which results from chronic insanity.* This disease conducts its victims to the tomb by a series of changes, as secondary and subordinate to that of the brain as the colliquative diarrhœa that closes a case of consumption; and, *in the latter instance, to say that the patient dies of diarrhœa would convey as false a representation of the fact,*

*as to say, of many of those who die insane, that their death is caused by diarrhœa, or marasmus, or exhaustion, because one or the other of these disorders happened to be the last obvious member of a series of morbid changes, the first, most efficient and characteristic of which had its seat in the brain.*"

In Rhode Island, as in New Hampshire, there are no sufficient laws authorizing the commitment of the insane to public hospitals. Dr. Ray thus writes upon the subject:—

"Our appropriate duties are performed rather by sufferance of public sentiment than any sanction of law, and thus we constantly lie at the mercy of excited passion and prejudice. The actual practice is, for those who stand in the nearest relation to the insane person, to place him in charge of an institution, and give the necessary obligations for his support. No one, certainly, can deny that this is right and proper, and in many cases it meets every practical requisite. The person is correctly considered insane, and he quietly submits to the measure. On recovery, he recognises its propriety and gives it his grateful approval. But in cases of a doubtful character, there should be a provision for some authoritative judgment, and especially in that class of cases where the person regards not only the deprivation of his liberty as the grossest outrage upon his rights, but is in a position, sooner or later, to seek redress for his fancied injuries. The probability of being involved in litigation would often induce one to forbear to interfere, even while every other consideration called for his interference. At any rate, the law, whatever it is, should be clearly defined, and should meet the difficulties experienced in the exceptional cases. The common law sanctions no confinement of the insane, except on the score of their safety, or that of society; and our statutes are silent upon the subject. For any other purpose, the measure is at the peril of those who seek it. True, it seems almost incredible that people should be punished for doing what common sense and common humanity prompted them to do; but it has happened, and may happen again, that an insane person believing, or affecting to believe, that his confinement was grossly unjust, though it resulted in his partial recovery, has resorted to the law for redress; and, by setting up false issues, and making artful appeals to the popular sympathies, has succeeded in convincing a jury that he was a much injured man, and obtained from them a verdict of vindictive damages."

The report contains some very reasonable remarks upon the very unreasonable requirements of many people, in regard to the treatment of their friends by the officers and their assistants in public institutions. After mentioning the safeguards which are thrown around the patient, it continues: "In these facts will be found a guaranty against improper practices, and, upon a broad estimate of the ordinary rules of action that govern men, it would seem to be a sufficient one. But it would be as idle to suppose that, in some mysterious way, the moment people are entrusted with the charge of the insane, they become transformed from men and women into angels; as that, on the other hand, they should systematically pursue a course opposed by every influence around them. There are exceptions to all general rules; imperfection and short-coming are incident to everything human; and if, occasionally, an attendant should so far forget or disregard his obligations as to utter a harsh word, or use unnecessary force, no sensible man would consider the fact as enough to outweigh the numberless benefits conferred by these institutions. To expect that a young person, without any extraordinary moral endowments, or any special preparation for the duty, *can bear, day after day, and hour after hour, week in and week out, the incessant and systematic efforts of one whose power for mischief is only heightened by disease, to teaze and irritate him, and never lose his patience, is to expect a phenomenon not often witnessed in any other department of life.*"

We once knew of the reception into an asylum of a woman whose relatives would consider the insinuation that they were not *respectable* as an unwarrantable insult. Her sister was very particular in requesting that she might be treated with all possible kindness, tenderness, and consideration. In her subsequent visits to the institution she was never weary in the endeavour to ascertain whether this humane course was ever departed from. In a few months the patient, appearing to be incurable, was removed, at the request of this sister, who now assumed the care of her. Forty-eight hours had not elapsed, after the removal, before the two had a regular *set-to,* which would have honoured the combatants of the ring, with such fisticuffiing, scratching, and pulling of hair as is not to be seen every day in well-regulated and affectionate families.

6. The principal statistics of the Hartford Retreat for the Insane, for the year ending March 31st, 1849, are as follows:—

| | Men. | Women. | Total. |
|---|---|---|---|
| Patients at the beginning of the year . . . | 59 | 63 | 122 |
| „ admitted in course of the year . . | 49 | 84 | 133 |
| Whole number „ „ . . | 108 | 147 | 255 |
| Discharged „ „ . . | 47 | 75 | 122 |
| Remaining at end of the year . . . . | 61 | 72 | 133 |
| Of those discharged, there were cured . . . | 20 | 50 | 70 |
| Died . . . . . . . . . . | 7 | 5 | 12 |
| Daily average number for the year . . . . | .. | .. | 141 |

*Causes of Death.*—General debility, 3; marasmus, 2; exhaustion, 2; phthisis, 1; "disease of brain," 1; "disease of lungs," 1; erysipelas, 1; suicide, 1.

Dr. Butler relates the following case in this report; "A. B. of C. was brought into the institution in 184—. The following is the history of the case, as given by the intelligent gentleman who brought him to the Retreat. B. is 36 years of age, and has been insane twenty years. When young, he was considered, in point of intellect, quite equal to most boys of his age, and was fond of reading and of mathematical studies. From some unknown cause he became a violent maniac, destroying everything in his way, and dangerous. The family became afraid of him, and chained him in a room or pen, partitioned off from the stable, in the barn. He would tear his clothes and any bed-clothing provided for him, so that he would often be entirely naked, the coldest nights in winter, without appearing to suffer by exposure. His usual dress was nothing more than a coarse flannel frock, and without anything for a bed but loose straw. He remained in this state for years, when his father, becoming poor, called upon the town for help. The select-men went and found the man as described, and consulted with the father as to what should be done. Their conclusion was, that, if the father had kept him in a barn, it would not be improper for the keeper of the paupers to do the same.

"Accordingly, he was removed from place to place, as the paupers were changed, and kept as his father had kept him. He was generally fed as we feed swine, had nothing but his hands to feed himself with, and, as all his filth remained in his stable for many days, it was *a fearful job to attempt to clear it out,* as the saying was. He was in an out-building, and without fire, for the twelve or fifteen years that he was supported by the town. He was in a sitting posture so long that the cords of the legs contracted, so that his knees are drawn up to his breast, while his legs are drawn close to his body. He is entirely negligent of the calls of nature.

"He is now," continues the report, "in the Retreat, demented; is always cheerful and docile, takes his meals regularly, and is cleanly in his habits and person. His lower limbs are closely contracted upon his body, and he accomplishes locomotion, with a good deal of agility, by placing his hands on the floor and swinging his body along."

From the report for 1849, we glean the following items:—

| | Men. | Women. | Total. |
|---|---|---|---|
| Patients at the beginning of the year . . . | 61 | 72 | 133 |
| Admitted in course of the year . . . . . | 60 | 75 | 135 |
| Whole number . . . . . . . . . | 121 | 147 | 268 |
| Discharged . . . . . . . . . | 48 | 77 | 125 |
| Remaining April 1st, 1850 . . . . . | 73 | 70 | 143 |
| Of those discharged, there were cured . . . | 17 | 47 | 64 |
| Died . . . . . . . . . . | 17 | 13 | 30 |
| Daily average number for the year . . . | .. | .. | 143 |

"The number of recoveries is larger than that of any previous year excepting the last, and this number would have been increased by the addition of several cases from among those reported as more or less improved, but for their ill-advised and premature removal from the institution.

"During the months of August and September," says the report, "we were visited by the same malarious influence which pervaded the valley of the Connecticut, as well as most other sections of our country. Though spared by a merciful Providence, from the ravages of the cholera (not a case of which has ever occurred in the institution), we had a large number of cases of diarrhœa and dysentery. The former yielded readily to treatment in nearly every case, and was fatal in none; while the latter appeared in a very severe and malignant form, and was very difficult to control. It proved fatal in the cases of eight patients and one attendant. The whole number of cases among the patients was forty-seven; twenty-two females, of whom four died, and twenty-five males, of whom four, also, died. Among the attendants there were eight cases, of whom one died.

"At different times during the year or two past, an epidemic erysipelas has prevailed in this city and vicinity, in a form of unusual severity. With a single exception, in the month of July last, no cases have occurred in the Retreat until last January, during which, and the two following months, we had twelve cases, of whom six died. *All* of these were old and incurable cases, and, with a single exception, were the most infirm and debilitated patients in the institution.

"In addition to the preceding deaths from epidemic diseases, there have been fifteen others (about the usual proportion of preceding years), from the following diseases: two of consumption; three of general debility; two of general paralysis; and one, each, of apoplexy, old age, paralysis, marasmus, disease of the heart, chronic diarrhœa, and dropsy; and one of exhaustion, an acute case complicated with febrile disease, and aggravated by the fatigue of travelling—an unfit case for the institution."

| | Men. | Women. | Total. |
|---|---|---|---|
| According to the report for 1850, the number of patients at the beginning of the year was . | 73 | 70 | 143 |
| Admitted in the course of the year . . . | 56 | 72 | 128 |
| Whole number . . . . . . . . . | 129 | 142 | 271 |
| Discharged . . . . . . . . . | 57 | 57 | 114 |
| Remaining April 1st, 1851 . . . . . | 72 | 85 | 157 |
| Of those discharged, there were recovered . . | 25 | 34 | 59 |
| Died . . . . . . . . . . | 9 | 6 | 15 |
| Daily average number for the year . . . . | .. | .. | 151 |
| Seven years ago, the same average was . . . | .. | .. | 84 |

During that period, the annual admissions have increased from 80 to 128.

*Causes of Death.*—Dysentery, 4; exhaustion, 3; general debility, 2; apoplexy, 1; epilepsy, 1; old age, 1; suicide, 1; general paralysis, 1; erysipelas, 1.

"Twenty-six of our patients were removed from the Retreat during the year, in different stages of improvement. Some of them were slowly but surely recovering." In a general allusion to discharges of this kind, the report says:

"The history of some of these premature and ill-advised removals is very sad. Many have relapsed into an incurable state, while others remain half-crazed, or nervous invalids, and will probably remain so for life."

Much of the increase in the number of patients, during the last seven years, is stated to have been the result of an appropriation, by the State, for the support, at this institution, of such lunatics as are unable to bear the expense. This fund was 2000 dols. in 1842, but was increased to 5000 dols. in 1843, at which sum it is still continued.

For many years past, the Rev. T. H. Gallaudet, one of the purest philanthropists of the age, has been connected, as chaplain, with the Hartford Retreat. With intellectual powers far above mediocrity, a heart overflowing with kindness, and a spirit ever rejoicing in whatever may contribute to the improvement of the physical or moral condition of any portion of his fellow-men, he was adapted, in a degree almost without parallel, for the situation whose duties he so honourably, faithfully, and usefully fulfilled.

The annual report of the physician to this institution has generally been accompanied by one from Mr. Gallaudet. That which is before us is his last—for since its publication, the institution and the public have suffered the necessary loss resulting from his death. The following extracts from this report is worthy of attention.

"In appreciating the benefits of institutions for the insane, regard is too much had simply to the cure or relief which they afford; and their utility is too often measured only by the amount of good thus effected. This is, indeed, their great object; and if this alone is considered, they have claims upon public bounty and private benevolence, than which none can be greater among the various forms of suffering humanity.

"But the many *collateral* advantages of such institutions are apt to be overlooked. If conducted by wise and observing individuals, they furnish the means of shedding clearer light upon questions of deep and general interest connected with the philosophy of mind, and the reciprocal influence which the mind and the body have upon each other—the elements of moral science—the education and training of children and youth, both in families and schools—the laws of hereditary physiology—the preservation of health and reason—prison discipline—criminal jurisprudence—and the precautionary measures to be pursued, to guard against many of the ills of the flesh and of the spirit—and then, of diffusing this light for the benefit of the whole human family.

"Such institutions ought to feel their responsibility in these respects, and be so conducted as to meet this responsibility. The light which they get should not be hid under a bushel. They occupy a position which can fit them to take rank among the greatest benefactors of mankind. The ill which they can be instrumental in *preventing*, outnumber and *outweigh*, thousands of times, those which, having already taken place, they are privileged to mitigate or remove.

"My mind has been turned to this subject from noticing, after thirteen years' experience as chaplain of the Retreat, what *a school of practical wisdom* it may be, and often is, not only to those who are placed for a time under its care, but also to the relatives and friends. They learn much of themselves, and not a little, in this respect, which they never knew before, much from their companions in misfortune, and much which they could never get from books or the common intercourse of society.

"They get new and more correct views of human nature; of what they should live for, and of the means of preserving a sound mind in a sound body, without which the great ends of life can be but very imperfectly, if at all, accomplished."

7. The Report of the Bloomingdale Asylum for the Insane, for 1849, is the first issued by Dr. Charles H. Nichols, who succeeded Dr. Earl, as physician to the asylum, in the spring of the year mentioned.

| | Men. | Women. | Total. |
|---|---|---|---|
| The number of patients Dec. 31st, 1848, was . | 59 | 60 | 119 |
| Admitted in the course of the year . . . | 58 | 37 | 95 |
| Whole number ,, ,, ,, . . . | 117 | 97 | 214 |
| Discharged ,, ,, ,, . . . | 54 | 36 | 90 |
| Died ,, ,, ,, . . . | 13 | 8 | 21 |
| Remaining December 31st, 1849 . . . | 50 | 53 | 103 |
| Of those discharged, there were recovered . . | 26 | 18 | 44 |

*Causes of Death.*—Pulmonary consumption, 4; "typho-maniacal delirium," 2; "apoplectic symptoms, occasioned apparently by a sudden increase of old serous effusions into the inner-cranial cavities," 4; "gradual exhaustion preceded by dysentery," 3; gradual exhaustion attended with diarrhœa, 4; suicide, 2; cancer, 1; delirium tremens, 1.

"In the fact, that among at least fifty persons with constitutions in a state of general decay, and offering but little resistance to epidemic agents, *no case of cholera occurred*, and but few cases of dysentery, though the former disease prevailed to some extent, and the latter very generally in the neighbourhood, is abundant evidence, if any were needed, of the salubrity of the site of the asylum."

Seven of the recoveries were cases of *inebriety*. "In nearly every case of intemperance received here," says the report, "the habit has existed, either continuously or periodically, for many years; and the individual has suffered numerous attacks of delirium tremens, or other sickness arising from drink; and every means but prolonged restraint has been exhausted to induce him to forsake the path to destruction in which he has so far advanced, but in vain; and at last delirium and stolidity are the only varieties of mental condition known to his experience, and he is totally unable to protect his interests or his person.

"The habit of intemperance is usually entered upon with the consent of a free will, and generally deserves to be treated as a vice; but my observations are confirmatory of the belief of Esquirol, Ray, and others, that in the case just described, a pathological state of the brain has been gradually induced, to which the will is wholly or in part subject; and I think physicians and magistrates need not scruple to grant the lunacy warrant, which we require in every description of case received here."

The following remarks upon moral treatment are very correct; but the grand *practical* difficulty is, to find amusements in which all the patients, or even a very considerable number of them, can join "as equals, if not principals."

"It has struck me that there is a material difference in value of what may be termed, in reference to the individual under care, the *active* and the *passive* modes of *moral* treatment. An amusement, a lecture, or a religious exercise which a patient witnesses merely, will often attract his attention, and thus, in a greater or less degree, suspend those morbid modes of mental action it is our object to eradicate; but if he himself takes an active part in the exercise going forward, his interest is enlisted on more self-respecting, not to say ambitious grounds, and is therefore more awakening and absorbing; and, as he has become an actor in a scene which suffers more or less interruption when he ceases to perform his part, the healthy mental effort is necessarily deeper, less divided, and more confirmatory of itself. In devising amusements for our patients, therefore, I have given preference to those in which they could participate as equals, if not principals. As, for digestion, it is better to walk than to ride; to saw wood than to see it burn; so, for the substitution of sound for deranged cerebral action, it is better that the patient should himself execute even poor music than hear the best executed by another; that he should constitute eighth of a cotillon than be the mere spectator of a score; that he should read aloud to others than that he should be trusted to listen."

| | Men. | Women. | Total. |
|---|---|---|---|
| The report for 1850 states that the number of patients in the Asylum Dec. 31st, 1849, was | 50 | 53 | 103 |
| Admitted in course of the year | 51 | 46 | 97 |
| Whole number | 101 | 99 | 200 |
| Discharged | 39 | 33 | 72 |
| Died | 12 | 6 | 18 |
| Remaining December 31st, 1850 | 50 | 60 | 110 |
| Of those discharged, there were cured | 28 | 22 | 50 |

Of the cures, 10 were cases of uncontrollable inebriety.

*Causes of Death.*—*Paralysie générale*, 4; chronic melancholia, 4; epilepsy, 3; chronic mania, 2; anasarca, 1; phthisis, 1; ascites, 1; hemiplegia, 1; suicide, 1.

"We have had no epidemic and no acute disease, or, at least, none that proved fatal in its first stages. In those instances where a more specific cause is not assigned, death was the inevitable and awaited final issue of a gradual deterioration of the organism, consequent upon long-continued derangement and deficiency of innervation."

Our extracts from this report will be limited to a single additional one, corrective of an opinion very generally entertained among the people.

"It is often queried, whether the separation of insane persons with sensitive minds from the family circle and endearments, and their commitment to the care of strangers, will not be attended with a sort of shock to the nervous system, and thus greatly aggravate the mental distress and aberration, and whether it be suitable that a number of such persons should be associated in a greater or less degree; but I believe it to be the uniform opinion of those experienced in this speciality of the medical profession, that the injurious effects of removal to an asylum, sometimes apprehended, never occur; and that the association of the insane, if there be a proper classification, very often essentially promotes recovery, and is attended with no objections whatever. No points in the management of nervo-mental diseases are better settled than these, and, if necessary, are capable of copious and very conclusive illustration."

8. Dr. Brigham, the late distinguished superintendent of the New York State Asylum, died in the summer of 1849, and his place, during the remainder of the year, was filled by his principal assistant, Dr. George Cook, by whom the report before us was written.

| | Men. | Women. | Total. |
|---|---|---|---|
| Patients at the beginning of the year | 241 | 254 | 495 |
| Admitted in the course of the year | 192 | 170 | 362 |
| Whole number | 433 | 424 | 857 |
| Discharged | 207 | 201 | 408 |
| Remaining at the end of the year | 226 | 223 | 449 |
| Of those discharged, there were cured | 113 | 90 | 203 |
| Died | 35 | 34 | 69 |

"During the past summer," says the report, "while the epidemic cholera pervaded a large portion of our country, we, through the kindness of an overruling Providence, were spared from its ravages; and, with the exception of some cases of dysentery, in the months of August and September, the general health of our patients was good. But in the month of December last (1848), the asylum was visited by the smallpox, which continued to prevail amongst us for several weeks, and in a number of cases proved fatal. No person who came here had the disease at the time of admission, or, as far as we could learn, had come from a section of the country where it was prevalent. It made its appearance in the female division of the asylum, and the first case occurred in a patient who had been here about seven months." The first, second, third, and fourth cases were very mild; the fifth, in a patient who had been at the asylum several months, confluent and severe. When attacked, the patients were removed to the infirmary.

"Of four hundred and ninety patients who were in the house at the time, and who were more or less exposed, forty-eight took the disease; viz., twelve men and thirty-six women. Thirty-three had it in a mild form; of these, six were men and twenty-seven women. Fifteen had the confluent form, of whom six were men and nine women. Fourteen died in the course of the disease, or soon after its termination; viz., five men and nine women, of whom eleven died of the disease, and in the other three, death was only perhaps a little hastened by it." Besides the above, eight attendants had the disease, two of whom died.

The remaining fifty-five deaths were caused as follows: Dysentery, 14; meningitis, 7; consumption, 6; exhaustion following excitement, 5; general paralysis, 4; epilepsy, 3; marasmus, 2; diarrhœa, 2; pneumonitis, 2; ascites, 1; hydrothorax, 1; suicide, 1; puerperal fever, 1; "disease of spinal cord," 1; erysipelas, 1; apoplexy, 1; "serous diarrhœa," 1; old age, 1; "peritoneal inflammation from perforation of the intestines," 1.

The general system of moral treatment introduced by Dr. Brigham is still pursued. The tailor's shop appears to be no unimportant item in this system, as the report contains a list of no less than *four thousand six hundred and four* garments and articles of household furniture made in it during the year.

The officers of this institution have for several years taken particular pains to ascertain the number of suicides that occur within the State of New York. They think that "nearly all" are included in their tables, the totals of which are—for 1845, *seventy-four;* 1846, *sixty-four;* 1847, *one hundred and six;* 1848, *eighty-eight;* and for 1849, *sixty-two.*

The report for 1850 is the first issued by Dr. N. D. Benedict, the successor of Dr. Brigham. It is elaborate, and ably written.

| | Men. | Women. | Total. |
|---|---|---|---|
| Patients at the beginning of the year | 226 | 223 | 449 |
| Admitted in the course of the year | 185 | 182 | 367 |
| Whole number | 411 | 405 | 816 |
| Discharged | 209 | 178 | 387 |
| Remaining at the end of the year | 202 | 227 | 429 |
| Of those discharged, there were cured | 94 | 77 | 171 |
| Died | 34 | 17 | 51 |

*Causes of death.*—Chronic mania, 12; acute mania, 2; dysentery, 13; general paralysis, 3; erysipelas, 4; pleuritis, 2; phthisis pulmonalis, 2; diarrhœa, 2; operation for strangulated hernia, acute gastritis, typhus fever, acute dementia, aneurism of aorta, phagedæna, ascites, metro-peritonitis, strangulation, suicide, 1 each.

Of the deaths from chronic mania the report says: "These cases presented no evidences of organic disease; no inflammation, or results of inflammation, in any tissue or organ. For months before their dissolution the capillary circulation became extremely feeble, the secretions imperfect, the elaboration and appropriation of food defective, and consequent emaciation ensued. The whole train of morbid phenomena being referable to insanity, it seems proper to report them as dying of mania rather than of marasmus." We suspect, however, that such cases are, in most asylums, reported as deaths from marasmus.

"Thirteen died of dysentery, though it was at no time epidemic in the institution. We include, under this head, a form of disease very unlike dysentery of private practice and of general hospitals, but which we believe is very common in asylums, and which we do not recollect to have seen called by any other name. It occurs in chronic cases, whose powers of life have long been gradually sinking, and in recent cases, who have become much exhausted by protracted excitement. With premonitory symptoms, or exposure to known exciting causes, the patient is suddenly seized, and generally in the night, with bloody discharges, scanty and gelatinous, or, more frequently, copious and serous, with no heat of skin or abdomen, nor pain or thirst, or loss of appetite or strength. Death supervenes a few days after the attack. We have perceived but little benefits from remedies in this form of disease, the treatment for ordinary dysentery proving entirely nugatory."

There were twenty-three cases of erysipelas in the course of the year, mostly in the cold months, when the air of the halls was the most impure. "It is said of one of the New England hospitals, before infested with erysipelas, that after the introduction of a system of forced ventilation, this formidable disease entirely disappeared."

One of the cures reported was that of a man who had been insane upwards of six years, had been several years in the asylum, and long considered as demented and incurable. "He would stand for hours in strange postures, apparently without thought or feeling. Gradually he began to take notice of things around him, and to exercise. He resumed his trade, that of a tailor, and at length acquired his former dexterity and skill." This case furnishes another proof, not only of the importance of perseverance in the treatment of the insane, but also of the singularity of this wonderful and mysterious disease. By "perseverance in treatment" we mean the keeping of the chronic insane at institutions where the circumstances of their position furnish the greatest aid to a spontaneous or natural cure; for we presume that, in this case, *medical* treatment had long been abandoned. The case reminds us of one which once came under our observation. A lunatic had been under curative treatment until the hope of restoration was relinquished. He was pronounced incurable; a commission of lunacy was immediately appointed, his case legally investigated, and he was put under guardianship. *Within three weeks from that time he was perfectly well, and soon returned to his employment as clerk in a large mercantile establishment.*

In the treatment of acute mania, with violence, raving, and consequent exhaustion, Dr. B. employs seclusion, hot baths with cold applications to the head, and free evacuation of the bowels. "*In no case*," says he, "*have we found local or general bleedings admissible; but, on the contrary, nutritious diet and brandy-punch are generally demanded.*"

The physician by force, in Molière's "*Médecin Malgré Lui*," speaks of the stomach as being situated upon the right side, and the liver upon the left. An interlocutor seems puzzled by this asserted position of the viscera, and mentions his impression that the stomach is on the left side and the liver on the right. Hereupon the physician by force acknowledges that, *formerly*, such was their position, but very sagely adds, "*nous autres médecins, nous avons changé tout cela.*" With much more truth may it be asserted, in regard to the treatment of acute mania, as recommended by Rush, and as generally practised in this country until within a comparatively few years, "*nous avons changé tout cela.*" This change has taken place, not at the Utica Asylum alone, but at all, or nearly all, the institutions for the insane in the United States.

"Of moral, or perhaps more correctly, *immoral* insanity," says the report, "nine cases have been under our care, two of whom have been admitted within the last year. These cases present the various forms of derangement, from the mere rascally little sinner (two were lads) up to the most aggravated form of the genuine disease. We have an idea that a remedy, not much known to modern science, but in vogue in the days of Solomon, commenced early and faithfully persevered with, would have been eminently successful in preventing the development of the disease, or, at least, arrested its progress before its full establishment. One of our patients is the exact counterpart, if not the identical fellow seen by Mr. George Combe, in the Dublin Lunatic Asylum, who exhibits a total want of moral feeling and principle, yet possesses intelligence, ingenuity, and plausibility. He has been a scourge to his family from childhood; was sent to the army to get rid of him, from which he was turned out as an incorrigible villain, always fighting and getting drunk, for which he was repeatedly flogged. By seclusion, he becomes so savage as to render the task of entering his room and supplying his wants by no means enviable; and when at large, he often assaults those around him. His chief employments are eating and fighting; and although he is constantly endeavouring to '*get out of these barracks*,' he seems to have no particular object in view but the more free indulgence of these propensities. In all but this one case, moral treatment alone has accomplished our object; but on him little moral influence can be exerted. By the aid of nauseating remedies, and purgatives frequently administered, we are enabled, in some degree, to control him. Blisters and setons to the back of his neck are now being tried."

The physicians to insane hospitals generally acknowledge their tables of the "causes of insanity" to be comparatively valueless. That they are so, we have a striking proof in the report before us. Of the *two thousand three hundred and seventy-six* patients admitted previously to 1849, only *nineteen*, or *four-fifths* of *one per cent.* are reported as having originated from masturbation; while of *three hundred and sixty-seven*, received in the course of the year mentioned, *fifty-three*, or more than *fourteen per cent.*, are attributed to that cause. Now, no reasonable man can believe that both of these statistical items can be true. Whence is the error? In the fact, undoubtedly, that they were reported, the former by one physician and the latter by another;—by two men, who, although they may have been equal in talent, learning, and skill, may have *favoured different theories;* or the one may have been somewhat more thorough in his investigations than the other.

"Frequently," writes Dr. B., "the patient himself can give the most satisfactory cause of his insanity, and often the very opposite to that attributed by his friends. This is especially true of masturbators, whose insanity is looked upon by friends as caused by 'religious anxiety,' because the first evidence of it noticed was an extraordinary anxiety about their salvation; an inordinate fear of future punishment; or abandoning all occupation but that of reading; or holding a Bible as if reading; or praying; or mumbling incoherent sentences, in an attitude of prayer, at improper times and places; or 'trying to tell his experience' in a religious meeting; or joining in and going to great lengths in the excitement of protracted religious meetings, or in such like acts. Another class, frequently placed under the head of 'religious anxiety,' are religious monomaniacs, whose insanity is undoubtedly referable to dyspepsia, habitual indigestion, and constipation, and the injudicious use of remedies for these diseases."

In the treatment of masturbation, "we rely mainly on mechanical restraint and aphrodisiac medicines. The combination we prefer is that of conium, camphor, and belladonna; and we think we have indubitable evidence of its power. We sometimes prescribe blisters and cold baths with advantage."

Although we have exceeded our usual limits in the notice of this report, we cannot leave it without laying before our readers the following extract:—

"Of the 816 patients in the institution, during the past year, the suicidal propensity existed in 66—22 males and 44 females. There were 28—21 females and 7 males—in the house at one time. In 20 of these 21 females the propensity was intense. To have at one time under care twenty-eight persons bent upon destroying themselves, is a burden which they alone know who bear it, increased by the necessity of carrying, at all times, amid surrounding sadness, a cheerful countenance over a heavy heart. The successful attempt at self-destruction, before reported, was made on the 12th of July, by a female patient of our most intelligent class. Her melancholy end became known to her companions, with whom she was a favourite, and, on the following day, two other patients on the same hall were overheard devising a plan for their own death. About this time, the suicidal propensity prevailed extensively, and seemed to be epidemic. There were admitted, during the month of July, the large number of forty-four patients, from different portions of the State,

*nineteen* of whom were suicidal. Several of these had attempted suicide immediately previous to admission. Two patients, *who had long been in the house, and never exhibited suicidal propensities,* attempted it during the month (on the 13th), *though they had no knowledge of the violent death that had occurred in another portion of the building.* On the 17th, a patient, believed to be entirely ignorant of all that had occurred previously, attempted strangulation, and continued to repeat the attempt until restrained by mechanical means. On the 20th, a patient tried to open a vein in her neck; and, on the 22nd, another, who knew of the suicide, and was no doubt influenced by it, attempted her destruction.

"From the 14th of July, fourteen attempts were made by eight different persons; and twelve others, in whom the propensity was strong, required constant observation. The suicidal epidemic prevailed from the 12th to the end of July, after which time it gradually subsided, and left the minds of most of the patients."

| | |
|---|---|
| The whole number of patients admitted since the opening of the asylum, is | 2743 |
| Of whom there have been discharged cured | 1188 |
| Died | 320 |

| | Men. | Women. | Total. |
|---|---|---|---|
| 9. The number of patients at the asylum on Blackwell's Island, New York, Jan. 1st, 1849, was | 187 | 250 | 437 |
| Admitted in the course of the year | 229 | 230 | 459 |
| Whole number | 416 | 480 | 896 |
| Discharged | 145 | 138 | 283 |
| Died | 85 | 127 | 212 |
| Remaining January 1st, 1850 | 186 | 215 | 401 |
| Of those discharged, there were cured (from insanity) | .. | .. | 172 |

*Thirty-six* cases of delirium tremens, one of hysteria, and three of febrile delirium, also recovered.

*Causes of death.*—Cholera, 86; chronic diarrhœa, 38; diarrhœa, 10; dysentery, 4; consumption, 21; congestion of brain, 12; apoplexy, 5; epilepsy, 5; paralysis, 2; paralysie génerale, 3; typhoid fever, 8; delirium tremens, 3; erysipelas, 2; old age, 4; and of scrofula, scurvy, suicide, albuminuria, typhoid pneumonia, chronic peritonitis, softening of the brain, dropsy, and exhaustion from exposure to cold, before admission, 1 each.

There were more deaths in June and July than in the remaining ten months —a mortality caused by the prevalence of the cholera. The first case of this disease was on the 10th of June, when there were 577 persons in the establishment, of whom 497 were patients. Of the whole number, 148 were attacked, and 91 died. The greatest number of new attacks, on any day, was 15, on the 9th of July; the last attack was on the 26th of the same month. "The principal sufferers were those who were usually lying about upon the floor or benches, regardless of their situation, and, in some cases, addicted to filthy habits, resulting from their demented state. Their physical condition was impaired generally."

The subjoined table shows the duration of the disease, from the time of attack, in the 91 cases of death:—

6 died in from 3 to 6 hours; all were collapsed *ab initio.*
18 „ 6 to 12 hours; all were collapsed *ab initio.*
30 „ 12 to 20 hours; all were collapsed, apparently *ab initio.*
16 „ 20 to 30 hours; all collapsed from 4 to 12 hours after attack.
6 „ 30 to 48 hours; 5 collapsed, 1 partially collapsed.
4 died on the 3rd day; all partially collapsed, and died from prostration.
4 died on the 4th and 5th; 2 collapsed, 2 partially so; all died from consecutive fever.
7 died after the 5th; 3 collapsed, 2 partially so; all died from consecutive fever.

"In those who were not entirely demented, the intellectual powers were apparently improved during the severity of the disease; but, at its subsidence, the mind resumed its previous condition."

Of the 148 cases, there was neither diarrhœa nor vomiting in 1, no diarrhœa in 1, no vomiting in 5, and no cramps in 59. Diarrhœa, vomiting, and cramps occurred in 82, and complete collapse in 90. Premonitory symptoms were known to exist in 61, to be absent in 31; and there were 56 in regard to which this fact was unascertained.

"In the case in which vomiting and diarrhœa were absent, there were severe cramps in the extremities, and extreme collapse, death occurring in three hours, followed by strong muscular contractions. The patient in whom diarrhœa was absent had severe cramps in the extremities and abdomen, excessive vomiting and feeble pulse, but recovered. The five in whom vomiting was absent were collapsed directly after the commencement of the disease. In one, cramps were likewise absent. All died, in three, five, four, three, and sixteen hours respectively. Of the 59 cases in which cramps were absent, 13 were partially and 32 completely collapsed: 36 of this number died."

The term *collapse* is used here in reference to those cases alone in which the patient was pulseless.

The erection of a new "Lodge" for violent patients, and of a large addition to the principal building, has given to the patients of this institution the additional room which was so much needed; and, rendering the improved management the more effective, has been of no little assistance in elevating the establishment above the wretched condition which made it a "shame and a reproach" to a Christian community. "Less restraint," says Dr. Ranney, "has been requisite, and frequently it has not been necessary, during the day, to apply any restraining apparatus, *or even to confine a single patient in his room. The number of violent paroxysms, accidents, and attempts to commit suicide, has been lessened. At least one-third of the whole number of patients have been engaged in some species of labour.*"

Why, Dr. Ranney, people who visited your institution in 1846 would hardly know where they were should they call there again. At that time, one would have as soon looked for a library at the sources of the Nile, or among the Esquimaux, as at that asylum; but now the patients are supplied with "biography, history, geography, philosophy, theology, poetry, fiction," &c., and "free access to the reading-room has contributed much to the restoration of convalescents." That is as it should be. No more blessed resurrection has occurred within the limits of our experience.

In the report of the visiting physicians, Drs. Ogden and Williams, it is remarked, in reference to the cases of cholera, that "several patients refused to take medicine, and those all died; while many in apparent extreme collapse recovered under medical treatment—an important fact, showing the fatality of the disease when left to the unassisted efforts of nature."

| | Men. | Women. | Total. |
|---|---|---|---|
| By the report for 1850, it appears that the number of patients on the 1st of January was | 186 | 215 | 401 |
| Admitted in the course of the year | 195 | 196 | 391 |
| Whole number | 381 | 411 | 792 |
| Discharged | 138 | 113 | 251 |
| Died | 43 | 34 | 77 |
| Remaining December 31st | 200 | 264 | 464 |
| Of those discharged, there were cured | .. | .. | 179 |

Among the cures were 25 cases of delirium tremens.

*Causes of death.*—Consumption, 23; general debility, 20; paralysis, 6; paralysie générale, 5; congestion of the brain, 5; epilepsy, 2; apoplexy, 2; dropsy, 3; stomatitis, 2; suicide, 2; inflammation of the brain, diabetes, empyema, lumbar abscess, erysipelas, chronic diarrhœa, and old age, 1 each.

The proportion of deaths, upon admissions, was four per cent. less than in 1848, and ten per cent. less than in any other year; that of recoveries was two per cent. greater than in 1848; and ten per cent. greater than in any previous year. Such are the expected, because the legitimate, results of the improved and still improving condition of the asylum.

From motives of "economy"—whether *domestic* or *political* we cannot assert, though, judging from the management of some of the institutions upon Blackwell's Island, while they were under the government of the common council of the city, we should strongly suspect it to be the latter—the convicts of the penitentiary have been employed as domestics and attendants at this establishment. Some of the results of this system are thus alluded to in the report:—

"The prisoners not only steal the clothing of the patients, but anything else of value that falls in their reach. As an illustration, the following case may be mentioned, *as one from a great number of cases of a similar character.* A few years ago, a young lady, who had been insane for some time, was admitted, and, although partially demented, her self-esteem was gratified by the possession of a beautiful head of hair. The morning after admission it was observed that her head was completely shorn, and, after a long examination, the ringlets so highly valued were found in the possession of a prison aid in the hall, who had committed the theft for the purpose of selling them to a peruke-maker."

The correction of this evil, by hiring suitable attendants, has been commenced, and will, undoubtedly, be completed before long. Various improvements, both within doors and without, were made in the course of the year. Among the former is the allowance, "*for the first time,*" to the patients, of knives and forks in several of the halls. One of the best evidences of improvement, to persons who know the former condition of this asylum, is found in the gardener's report, where it is stated that an aggregate of 2779 days' labour was performed by the patients, between the 26th of May and the 31st of December. They raised *twenty thousand* cabbages, and other vegetables in proportion.

The visiting physicians, in their report, say that the number of pauper lunatics in New York city, on the 1st of September, 1834, was 116; whereas, on the 1st of January, 1851, it was 464. "Estimating the future increase from these data, the city and county of New York will, fifteen years hence, have more than a thousand lunatics to be supported at the public charge."

They suggest various improvements, which, if adopted and effected, will render this institution one of the best of its kind. At the close of the report, Dr. Williams resigns the place of attending physician.

10. From the report for 1849, of Dr. Buttolph, of the New Jersey State Lunatic Asylum, we extract the following statistics:—

| | Men. | Women. | Total. |
|---|---|---|---|
| Patients in the Asylum January 1st, 1849 | 46 | 37 | 83 |
| Admitted in the course of the year | 55 | 41 | 96 |
| Whole number | 101 | 78 | 179 |
| Discharged | 39 | 30 | 69 |
| Remaining January 1st, 1850 | 62 | 48 | 110 |
| Of those discharged, there were cured | 24 | 20 | 44 |
| Died | 4 | 5 | 9 |

*Causes of death.*—Exhaustion, 5; consumption, 2; chronic diarrhœa, 2.

"During the prevalence of the cholera in neighbouring places, a marked epidemic tendency to affections of the digestive organs prevailed in the institution; but no death, or very alarming sickness of that character, occurred."

The cure of a woman, insane more than *eighteen* years, and that of a man whose disease had existed upwards of six years, are reported. Of the former, Dr. B. says, "No expectation was entertained of her recovery by her friends or the officers of the institution; and it must be regarded as a very unusual exception to the general rule of success, and to be attributed rather to a happy and rare effort of nature, than to the course of treatment adopted, which, at best, could only be considered as having favoured such a result." Of the latter he remarks, that in the recovery of the patient he was "also agreeably surprised, and could scarcely believe that a permanent cure had been effected, until some months of careful observation of his mental state had established the fact."

Now, granting that both of these remarkable cures were, as is suggested of the first, the effect of a "happy effort of nature," the question may still be asked, If it be likely that the "happy effort" would have been crowned with such success, had the patients not been taken to an asylum? We think it would not. Nature wanted just such assistance as can be and is rendered by a well-conducted institution.

The principal part of this report is devoted to a detailed account of the management of the institution, its daily domestic duties, &c. &c.

We proceed to the report for 1850.

| | Men. | Women. | Total. |
|---|---|---|---|
| Patients at the beginning of the year . . . | 62 | 48 | 110 |
| Admitted in the course of the year . . . | 52 | 58 | 110 |
| Whole number . . . . . . . . | 114 | 106 | 220 |
| Discharged . . . . . . . . | 28 | 30 | 58 |
| Remaining January 1st, 1851 . . . . | 86 | 76 | 162 |
| Of those discharged, there were cured . . . | 15 | 17 | 32 |
| Died . . . . . . . . . | 6 | 4 | 10 |

*Causes of Death.*—Apoplexy, 3; consumption, 2; exhaustion, 2; chronic mania, 1.

Dr. Buttolph makes the following remarks upon treatment:—

"We use medicine sparingly, being influenced somewhat by the opposition that many insane have to taking it; but more especially by the fact, that a physiological treatment is frequently quite as salutary as medical, and vastly more agreeable to the patient. Under the head of mental and moral treatment we include all those means and influences that can be brought to bear upon a person through the medium of the mind and feelings. Thus, the removal of a person from home, and the associations with which their excited, depressed, or perverted feelings have arisen, is often nearly all that is required to restore the healthy balance of the faculties. But, in addition to the effect of separation from irritating causes at home, the new scenes, regulations, employments, amusements, and, indeed, the petty inconveniences and even annoyances met with in an institution, often have the effect, insensibly, to withdraw the attention of the patient from subjects upon which he has dwelt to his injury. Hence, treatment in an asylum is usually more successful than in private, and, as a general rule, is to be recommended. Occasionally, however, cases arise in which the question of removal from home can only be properly settled by an experienced medical adviser, or by resort to the experiment of change."

After mentioning some improvements in the means of heating the buildings, which is done by steam, the report continues as follows: "As now working, we may safely challenge the world to produce another apparatus so perfect in the arrangement of its details, and so satisfactory in its results."

Dr. B. recommends an enlargement of the building by the addition of two wings, one on either extremity of the present structure, and each to accommodate thirty-eight patients.

11. Dr. Kirkbride, in the report of the Pennsylvania Hospital for the Insane, for 1849, says, that the institution was full at the commencement of the year, and continued so until its close. The average number of male patients was 110, and of females 99. An additional wing, for the accommodation of twenty more women, was constructed in the course of the year. "When the institution was opened, in 1841, it offered accommodations for only 140 patients and their attendants. Since then, additions have been put up, at various times, which will now contain 80 patients with their attendants, making four new classes of each sex, and giving two fine infirmaries, and a great variety of fixtures and arrangements, of immense importance to the comfort of all, but which were scarce thought of in the commencement of the main building."

The recent additions are heated by steam. "The character of the warm air from a steam or mild hot-water apparatus," says Dr. K., "is so entirely different, and so incomparably more pleasant than that from the common hot-air furnace—its neatness, avoiding, as it does, all dust, dirt, or gas in the rooms, is so striking, and—after the first cost of the fixtures—its economy is so evident, that I feel no hesitation in saying that no one, who has had an opportunity of testing its advantages, will, with our present knowledge, be willing to see any other system than one of these adopted in any building like a hospital, whether for the ordinary sick or for the insane."

| | |
|---|---|
| Patients at the beginning of the year | 200 |
| Admitted in the course of the year | 208 |
| Whole number | 408 |
| Discharged | 187 |
| Remaining at the end of the year | 221 |
| Of those discharged, there were cured | 104 |
| Died | 19 |

*Causes of Death.*—Pulmonary consumption, 5; apoplexy, 2; congestion of brain, 1; acute mania, 4; chronic inflammation of the intestines, 2; chronic organic disease of brain, 1; exhaustion from high excitement, 2; bronchitis, 1; pericarditis, 1.

Upon the approach of the cholera, "every reasonable precaution was taken to avoid the exciting causes of that disease. When it is recollected that the epidemic prevailed for some time in our vicinity, and that a public institution within sight of us lost no less than two hundred and twenty-nine of its residents, of whom seventy were insane, we must all feel that we have cause for devout thankfulness to a protecting Providence that I am able to record the fact, that not only was there not a single case of cholera in our household, but that there was no serious acute sickness of any kind, and less general indisposition than is commonly prevalent in the institution and its vicinity."

"The museum and reading-room, put up by the patients and friends of the institution, and presented to it as a Christmas offering, last year, has been in daily use, and has proved a source of great enjoyment to a large number of the inmates of the hospital." The report is ornamented with beautifully executed wood-cuts, representing the exterior and the interior of this building, so valuable an acquisition to the inmates of the establishment. There are also, similar views of the "Patients' Cottage" and the "Ladies' Summer House."

Although the facilities furnished, at this institution, for the moral treatment of its patients, are not exceeded, perhaps not equalled, at any similar establishment in the country, yet Dr. Kirkbride, in his untiring philanthropy and his characteristic striving for *the perfect,* looks forward to more. "The treatment of the insane," says he, "has been gradually improved, till many persons believe that little more is to be accomplished. This, however, is a serious error, and ought to be disavowed by all who are familiar with the wants of the insane. Many highly important means of treatment are still to be procured, or their use widely extended, and nothing but an absolute want of pecuniary ability ought to prevent a much greater degree of efficiency than has ever yet been attained. Conspicuous among these means are the various measures connected with the direct mental treatment of the patient—important in all cases, even in those apparently the most hopeless—but indispensable for many whose diseases assume forms that make them peculiarly interesting."

The report for 1850 is the tenth issued by the institution and by Dr. Kirkbride. It contains so large an amount of valuable matter that, although there will be no difficulty in beginning to make extracts, yet we fear that it will not be so easy a matter to decide when and where to stop.

| | |
|---|---|
| Patients at the beginning of the year | 221 |
| Admitted in the course of the year | 207 |
| Whole number | 428 |
| Daily average number | 219 |
| Discharged | 215 |
| Remaining at the end of the year | 213 |
| Of those discharged, there were cured | 106 |
| Died | 27 |

*Causes of Death.*—Pulmonary consumption, 5; acute mania, 5; inflammation of brain, 3; apoplexy, 2; dysentery, 2; general paralysis, 2; softening of the brain, 2; exhaustion following excitement, 1; chronic uterine disease, 1; epilepsy, 1; purpura, 1; disease of heart, 1; old age, 1.

Six of the patients died within two weeks from the time of admission.

"While simple insanity does not often produce death, it unquestionably tends to lessen the average duration of life, by rendering the individuals labouring under it less able to resist attacks of acute disease, by the difficulty often experienced in discovering sickness in its commencement, and by the resistance offered to the adoption of a proper course of treatment. There is, however, an acute form of insanity which does often cause death by a kind of exhaustion induced by the combined operation of long-continued mental excitement, want of sleep, and refusal of food. To distinguish these cases from ordinary insanity, to which they have little resemblance, the mode in which death has appeared to be caused has been inserted in the table. When acute disease of the brain has been referred to, it is intended rather to designate active inflammation of that organ than insanity."

After treating of the utility derived from the farm and garden, the workshop and mechanical department, and the museum and reading-room—the last of which has been found so useful that another, so that there shall be one for each sex, is desired—the report continues as follows:—

"During nine months of the past year, the course of lectures and entertainments in the lecture-room *was kept up regularly three times a week*, to the great gratification and benefit of the patients and those employed in their care. I have no knowledge of such a course having been regularly continued for so long a period in any other institution, and it was interrupted only on account of the hot weather rendering the room uncomfortable for so large an audience. During this intermission, on several evenings of the week, the patients were entertained in other modes, on the lawn in front of the main building.

"The practice of daily reading, by the teachers, to the patients in the different wards, especially those devoted to the more excitable class of patients, has been continued with marked good effect.

"The entertainments in the lecture-room have almost entirely done away with the social parties for both sexes that, in the earlier days of the institution, were frequently given, and the effects of the former have been found, upon the whole, to be much more satisfactory. Frequent sewing parties are still held by the matron, among the ladies of the different wards, and a grand entertainment, for all in the house, is always expected on Christmas eve, preparatory to the special dinner given on the following day."

A new feature has been added to the mental treatment, by the establishment of a library *in each ward*, of which there are sixteen. These libraries contain eleven hundred volumes. "A trial of three months has already been made with these books, and the result is most gratifying. The expressions of satisfaction, and of the benefit derived from them, by the most intelligent patients, is of itself sufficient to show their great importance, and but three volumes, of little value, are reported to me as having been injured."

We now come to that part of the report which has reference to the whole period of the existence of the institution. This is introduced by some, in our opinion, very just remarks upon statistics, from which we shall extract the most important passages.

"The value of statistical tables, on any subject, must, in a great measure, depend upon the competency of the observer, and the care that is exercised in their preparation; *but the fact that there are some inherent difficulties in the case can scarcely be deemed a sufficient reason for making no attempt to overcome them, or not approaching as near as possible to absolute certainty.* There seems to be no sound reason why the statistics of insanity may not possess as much certainty as those of most other maladies. Notwithstanding the false deductions made by those who have carelessly analyzed these reports and tables, it must still be acknowledged that this evil will be likely to correct itself; and it cannot be denied that, with all their defects, the general circulation of hospital reports, containing the results of judicious treatment, has done more to enlighten the public mind in reference to insanity, to stimulate and give proper direction to the efforts of philanthropists, and eventually lead to a liberal provision for the wants of the insane generally, than all other means combined.

"One great error, often committed in reference to the statistics of hospitals for the insane, has been in using those from different institutions as a basis of comparison, without alluding to the varied character of these establishments, the kind of patients received, in regard to their curability and general health, the different modes prescribed for their admission, the authority to detain them for treatment without regard to the caprices of friends, and various other circumstances having an important bearing upon the results, and without a full knowledge of and allowance for which, all comparisons are perfectly useless.

"Of all the medical subjects that can be tabulated, the number is exceedingly small in which the statements are not, to some extent, matters of opinion, and this latitude is as allowable in reference to insanity as to any other malady."

| | Men. | Women. | Total. |
|---|---|---|---|
| Whole number of patients admitted . . . | 999 | 807 | 1806 |
| ,, ,, ,, ,, discharged cured . | 466 | 377 | 843 |
| ,, ,, of deaths . . . . . | 104 | 72 | 176 |

"The number of males in the institution has generally preponderated (over that of females); but not universally. In nearly every year at some period, the number of the sexes has been equal, and, at other times, there have been more females than males."

The attention of those who have made themselves familiar with the reports of our institutions for the insane, during the last ten or fifteen years, must have been arrested by the fact that the number of females, not only absolute but relative to that of males, in those establishments, has been gradually increasing. While this truth indicates greater public confidence in the utility and the management of the hospitals, it throws a doubt upon what was believed to be a fact in former years—that the number of insane men in this country exceeds that of insane women.

"Among the cases embraced in this report, by far the most prevalent cause of insanity has been ill-health of various kinds, and in about the same proportion in both sexes. Intemperance is set down as the *direct* cause, in 106 (out of 1806) patients, of whom 97 were men and 9 women. This, however, is far from showing its real influence in the production of the disease. It tells nothing of its effect on others, nothing of the blighted hopes, the losses of property and character, the domestic difficulties and the mental anxiety, deep and depressing, which follow in its train and owe their origin to its existence. Loss of property, directly or indirectly, is a not infrequent cause of insanity, affecting men much more than women; while domestic difficulties are a vastly more common cause of its existence among females than males."

Fifteen cases, ten men and five women, were attributed to fright. They "were well marked, and resulted directly from that cause." After mentioning various other causes, the report continues: "Two cases in men and five in women, are reported as caused by the use of opium; and four in men, by the use of tobacco. Opium is much more used by females than males, and its effects upon the mind, no less than upon the body, are of a most injurious character. The use of tobacco has, in many individuals, a most striking effect on the nervous system, and its general use in the community is productive of more serious effects than is commonly supposed. I have never seen anything more than a temporary annoyance result from its entire discontinuance, *and by that course alone the complete re-establishment of impaired health has often been produced.*"

Some physicians report the loss of sleep as a not infrequent cause of mental derangement. Dr. K. gives no case from this origin, as he has found that the loss of sleep arose from some antecedent cause, or was the *effect* of the insanity.

When the physicians to asylums have deprecated the practice of general bleeding in insanity, they have frequently been met by the argument that *they* do not receive patients until the acute stage has passed away, and that, consequently, their authority for the treatment of that early stage cannot be valid. Of the 1806 cases reported by Dr. Kirkbride, in no less than 913 the disease was of less than three months' duration. It is not unreasonable to suppose that a large number of these had not existed two months, and many of them not *one.* Now, where are there any *ten* physicians, in general practice, in one city or vicinity, whose combined experience in the treatment of even *acute* insanity is equal to that of Dr. Kirkbride's? And yet we venture the assertion—and we call upon the Dr. to correct us, if we are in error—that, in all these 913 cases, Dr. K. has not practised venesection, *for insanity,* in a single instance. He may have done it for apoplexy, or congestion of the brain; but for mania, melancholia, or any of the maladies generally included under the name insanity, we presume to say *never.*

But perhaps we shall be referred to the authority of Dr. Rush, whose work on mental disorders is the only one generally known in this country. If so, we have two answers and another authority to offer. First: If, in the time of Dr. Rush, venesection actually was the best treatment for insanity, it does not necessarily follow that it is so now. Second: We consider the authority of Dr. Kirkbride, in the treatment of this disease, as of far greater weight than that of Dr. Rush, and that simply because we believe his experience to have been greater. Now for our authority; and it comes from a high source, the centre of London. In the early part of the present century, the system of treatment at Bethlem Hospital for the insane "consisted of *bleeding, purging, and vomiting, in the spring months. A certain day was appointed on which the patients were bled; another when they were purged; another when they were vomited. They were bled in May, and again in June,* THE PRECISE TIME DEPENDING ON THE WEATHER. The two authorities are contemporaneous. The latter is from an hospital so elevated in its position, that it is the only one, in the whole kingdom of Great Britain and Ireland, which is exempted from the inspection and surveillance of the Commissioners of Lunacy, and whose physicians, it must therefore be presumed, are among the most eminent in London.

But the physicians of probably nineteen-twentieths of the institutions for the insane, not only in America and Great Britain, but in France, Prussia, and Austria, condemn the practice of general bleeding, in insanity, unless it be in rare and exceptional cases.

Dr. Kirkbride has found mania to be the most curable of any of the specific forms of insanity. Next, in this respect, follows melancholia. Monomania occupies the third place; and the least proportion of cures—fifteen in two hundred and twenty-one—was in dementia.

We close our notice of this report with an extract relating to the provision for the insane in Pennsylvania.

"It is now just about a century since the Pennsylvania Hospital, the pioneer institution for the insane in America, was incorporated by the Provincial Assembly, and opened for the reception of patients. With the exception of the Friends' Asylum, at Frankford, established in 1817, and an Insane Department of the Philadelphia Almshouse, at Blockley (which, a few years since, for the first time, took rank as a curative establishment), the Pennsylvania Hospital has been the only institution in the State to which any class of her citizens could resort for the treatment of insanity, and it was, strictly, the only one which offered relief from this malady, without cost, to the indigent of Pennsylvania.

"From the foundation of the Pennsylvania Hospital, in 1751, to the present time, 6062 insane persons have been admitted and treated in its wards. Of these, more than 1000 were poor, who received every care and attention without charge of any kind, and of whom a large proportion were restored to their families in perfect health, and many others in various states of improvement; the number of this class under treatment being limited only by the income of the institution.

"It will be a fitting commemoration of the services rendered by a private charity to all classes of the insane, but especially to the indigent insane of Pennsylvania, during a whole century, that, exactly at the end of that period, our noble Commonwealth will have prepared and put in operation a State Institution,* intended to afford relief to all her citizens who labour under loss of reason, and which, with a judicious organization, and fostered by liberal and enlightened legislation on the part of the government, cannot fail to spread blessings of inestimable value throughout the community.

"When the new institution is in operation, about one thousand insane patients will be comfortably provided for in the State, and, except an hospital in its western part, Pennsylvania will require no material extension of the accommodations for her insane, for many years, although important improvements will be desirable in all the existing institutions."

12. The official year, of the "Asylum for the Relief of Persons deprived of the use of their Reason," at Frankford, Pa., commences with the 1st of March.

| | Men. | Women. | Total. |
|---|---|---|---|
| Number of patients March 1st, 1848 | 24 | 31 | 55 |
| Admitted in the course of the year | 19 | 19 | 38 |
| Whole number | 43 | 50 | 93 |
| Discharged | .. | .. | 46 |
| Remaining March 1st, 1849 | .. | .. | 47 |
| Of those discharged, there were cured | .. | .. | 25 |
| Died | .. | .. | 5 |

*Causes of Death.*—Effects of long excitement, 1; organic disease of the brain, 1; old age, 1; tumour on the brain, 1; acute mania, 1.

Schools and lectures constitute a part of the moral or mental treatment of the patients. "The experience of the past year," says the report, "confirms the opinion heretofore expressed, of the great utility of mental occupation, as well as bodily labour, in the curative treatment of the insane; and also its great importance in promoting the comfort and well-being of those who are incurable. It is not to be expected that the latter class should be capable of

* At Harrisburg. It is now in operation.

making much advance in learning, though their mental powers are certainly strengthened, and more developed by being brought into use, and stimulated by exercise; but, independent of this, important benefits result to them, from the efforts made to interest and employ their minds, inasmuch as they soon begin properly to appreciate the care and attention required to instruct them, and manifest their willingness to repay it by increased correctness of deportment."

In the course of the year, means of forced ventilation were introduced into some parts of the building, the old bath-rooms were improved, and two new ones arranged.

The leading statistics, from the report for 1849, are as follows:—

| | Men. | Women. | Total. |
|---|---|---|---|
| Patients at the beginning of the year | 24 | 23 | 47 |
| Admitted in the course of the year | 16 | 11 | 27 |
| Whole number | 40 | 34 | 74 |
| Discharged | .. | .. | 26 |
| Remaining at the end of the year | .. | .. | 48 |
| Of those discharged, there were cured | .. | .. | 14 |
| Died | .. | .. | 4 |

*Causes of Death.*—"Obstruction of the bowels," 1; acute bronchitis, 1; typhoid fever, 1; suicide, 1.

"Although the cholera prevailed at Frankford and in the vicinity of the asylum, yet the inmates of the institution were mercifully preserved from its fearful visitation; but, during the last summer and the first fall months, epidemic dysentery prevailed, to a considerable extent, among the patients and their attendants."

The report says that "a detailed description of the means that have been employed (in treatment), would be little more than a repetition of the matter of previous reports," and, consequently, no such detail is given. We find a similar idea expressed in the reports of several other institutions. Now, so far as our observation has extended, comparatively few people read the reports of asylums for the insane, other than physicians and those who have some near relative or friend suffering under mental alienation. Hence, a very large proportion of those readers is constantly changing. The new class of them are mostly ignorant of the modern method of treatment, and ought, as they generally wish, to be enlightened thereupon. It has, therefore, long been our opinion that each report of every institution should contain a description of the moral treatment, so full as to give a clear comprehension of it to a person previously without any knowledge upon the subject.

At or about the commencement of the official year for 1850-51, an important change was made in the organization of the Frankford Asylum, by making a physician its superintendent or principal officer. Dr. Joshua H. Worthington, who, for several years had been the resident physician, was appointed to the place. He is well qualified for the fulfilment of its duties.

| | |
|---|---|
| Patients at the beginning of the year | 48 |
| Admitted in the course of the year | 20 |
| Whole number | 68 |
| Discharged | 25 |
| Remaining March 1st, 1851 | 43 |
| Of those discharged, there were cured | 12 |
| Died | 2 |

"In general," says Dr. Worthington, "the time required for the cure of any case of insanity will depend upon the promptness or delay with which the patient is submitted to proper treatment. The earlier the treatment is commenced, the more speedy will be the recovery; and the reverse. We occasionally, however, meet with cases of long duration, in which the condition of the patient has been much neglected, or where the disease may have been kept up by improper treatment, which recover rapidly when placed under different circumstances. An instance of this kind was that of a young man from one of the interior counties of this State, who was discharged during the last year. He had been insane for two years previous to his admission, and, at the commencement of the attack, had attempted to take his own life by leaping into a well, and afterwards had been kept bound with chains. Under our care, he recovered in the course of a few months; and, during the period of nearly a year that has elapsed since his return home, he has continued entirely well, and been usefully employed in the management of a farm."

In regard to the curability of insanity, Dr. W. states, that, "in this institution, with the reception of all classes, and the disadvantage of premature removals, the per-centage of cures of recent cases, since 1842, is 72.25, there having been received, since that time, 191 cases of that description, of which 138 have been restored. If to this we add 10 per cent. as the probable loss sustained by premature removals, we shall have 82.25 per cent. which may be considered as nearly representing the proportion in which recent cases of insanity are curable. During the same period, 121 chronic cases have been admitted, 24 only of which, or 19.83 per cent. have been restored; the proportion of cures, on the whole number received in that period, being 51.92."

13. Dr. John Fonerden became connected with the Maryland Hospital in 1846; but no report, written by him, was published until the close of 1849. This report, therefore, contains the statistics of four years.

| | Men. | Women. | Total. |
|---|---|---|---|
| Patients at the hospital January 1st, 1846 | 58 | 49 | 107 |
| Admitted in the course of four years | 139 | 113 | 252 |
| Whole number | 197 | 162 | 359 |
| Discharged | 131 | 95 | 226 |
| Remaining December 31st, 1849 | 64 | 69 | 133 |
| Of those discharged, there were cured | 43 | 36 | 79 |
| Died | 40 | 17 | 57 |

"There were admitted, exclusive of the patients enumerated above, 107 private boarders affected with mania à potû. All of these were discharged recovered, except three who died. As asylums for the insane are not appropriate places for cases of this character, it will probably be discovered, in the progress of moral intelligence, that it is a proper function of the Temperance Societies to adopt the plan of building, on a farm near each of the principal cities, a suitable retreat; to be conducted, under the advice of a physician, by managers of mature age and discretion, who, having the promotion of temperance in view, and sufficient leisure, would aim, by their personal aid, to lead young men, after recovering from the dreadful malady, to love sobriety and usefulness of conduct."

"The number of recent cases of insanity admitted during the four years," continues the report, "was very small. Almost all the cases were of more than one year's duration before admission."

Dr. F. mentions the defects of the hospital, and the necessity of a "thorough reform." He evidently looks forward to a new architectural arrangement of the building, or to the erection of a new one, in a more suitable place. We hope that no considerations will induce the managers of that institution to decide upon the former course. Between the investment of a pretty large amount of funds in the attempt to make the present establishment what a hospital for the insane ought to be, and throwing the same sum into the river, there would be, in our opinion, but little choice.

In allusion to defective training, in early life, as a cause of mental disorder, the report closes with the following beautiful effusion of the heart of a father and, in the best and noblest sense of the term, a *man*:—

"How important is it, then, that childhood and youth should be gently led, by a patient and loving help, both in play and at pleasant work, into innocent habits of the mind, and, in agreement therewith, into bracing habits of the body. For, so far as such conjoined habits become identified with the physiological life, they will combat, triumphantly, many a hereditary peculiarity, mental and corporeal; and they will be strong in vital power to resist the invasion of disease. More than this; becoming, in due time, subservient to the religious principle, in its legitimate works of sincerity and justice, they will surely generate a purity of purpose in the discharge of domestic and all other duties; and thus, by exempting the mind from an abiding presence of selfish thought and inclination, they will be a safeguard against most of the secondary causes of disordered ideas and emotions, of incoherent speech and impulsive actions. So may the human mind, apart from the blighting power of unavoidable disease and accident, gradually work out its emancipation from the infirmities of a natural temperament; so can it earn the faculty of living in freedom according to reason."

Statistics from the report for 1850:—

| | Men. | Women. | Total. |
|---|---|---|---|
| Patients at the beginning of the year . . . . | 64 | 69 | 133 |
| Admitted in the course of the year . . . . | 25 | 15 | 40 |
| Whole number . . . . . . . . . | 89 | 84 | 173 |
| Discharged . . . . . . . . . | 21 | 11 | 32 |
| Remaining at the end of the year . . . . | 68 | 73 | 141 |
| Of those discharged, there were cured . . | 8 | 6 | 14 |
| Died . . . . . . . . . . | 5 | 1 | 6 |

Seven cases of mania à potû were also received, and discharged cured.

Dr. Fonerden calls the attention of the President and Board of Visitors to the necessity of providing additional accommodations for the insane, in the State of Maryland. The only argument adduced is the impossibility of receiving all the applicants at this institution. "It may now happen," says he, "that one or two months will elapse before another public patient can be received. In the mean time, urgent applications will continue to be made for the relief of the public and of families, and for the protection of the destitute insane, whose cases, in most of the counties, are dependent upon this institution for custodial arrangements. On the day of writing this, applications have been made for the admission of three patients at the expense of the counties."

